# Sphingosine-1-Phosphate Signaling in Immune Cells and Inflammation: Roles and Therapeutic Potential

**DOI:** 10.1155/2016/8606878

**Published:** 2016-02-07

**Authors:** Masayo Aoki, Hiroaki Aoki, Rajesh Ramanathan, Nitai C. Hait, Kazuaki Takabe

**Affiliations:** ^1^Division of Surgical Oncology, Department of Surgery, Virginia Commonwealth University School of Medicine and Massey Cancer Center, West Hospital 7-402, 1200 East Broad Street, P.O. Box 980011, Richmond, VA 23298-0011, USA; ^2^Department of Biochemistry & Molecular Biology, Virginia Commonwealth University School of Medicine and Massey Cancer Center, West Hospital 7-402, 1200 East Broad Street, P.O. Box 980011, Richmond, VA 23298-0011, USA; ^3^Breast Surgery, Roswell Park Cancer Institute, Elm & Carlton Streets, Buffalo, NY 14263, USA

## Abstract

Sphingosine-1-phosphate (S1P) is a bioactive sphingolipid metabolite involved in many critical cell processes. It is produced by the phosphorylation of sphingosine by sphingosine kinases (SphKs) and exported out of cells via transporters such as spinster homolog 2 (Spns2). S1P regulates diverse physiological processes by binding to specific G protein-binding receptors, S1P receptors (S1PRs) 1–5, through a process coined as “inside-out signaling.” The S1P concentration gradient between various tissues promotes S1PR1-dependent migration of T cells from secondary lymphoid organs into the lymphatic and blood circulation. S1P suppresses T cell egress from and promotes retention in inflamed peripheral tissues. S1PR1 in T and B cells as well as Spns2 in endothelial cells contributes to lymphocyte trafficking. FTY720 (Fingolimod) is a functional antagonist of S1PRs that induces systemic lymphopenia by suppression of lymphocyte egress from lymphoid organs. In this review, we summarize previous findings and new discoveries about the importance of S1P and S1PR signaling in the recruitment of immune cells and lymphocyte retention in inflamed tissues. We also discuss the role of S1P-S1PR1 axis in inflammatory diseases and wound healing.

## 1. Introduction

Sphingosine-1-phosphate (S1P) is a bioactive sphingolipid mediator involved in many physiological processes including angiogenesis and immune responses [1, 2]. S1P signaling has been found to be essential for vascular development, neurogenesis, and lymphocyte trafficking [3–5], as well as a second messenger during inflammation [6, 7]. Many of the actions of S1P in innate and adaptive immunity are mediated by its binding to five specific G protein-coupled receptors, S1P receptors (S1PRs) 1–5. To date, a number of S1P receptor modifying compounds have been developed [8]. FTY720 (Fingolimod, Gilenya, Novartis) is a functional antagonist of S1PR and was originally discovered by chemical modification of a natural product, myriocin. FTY720 and other S1PR modifying compounds have clarified that S1P is important for the recruitment of various types of inflammatory cells [9, 10]. In this review, we summarize current research findings on the functions of S1P in the recruitment of immune cells into inflamed tissues and discuss its role in inflammatory diseases and wound healing.

## 2. Sphingosine Kinases (SphKs) and S1P Signaling

S1P is a pleiotropic, bioactive, lipid metabolite of ceramide. Ceramide is the basic unit of sphingolipids and consists of a sphingosine attached to a long-chain fatty acyl group via its amino group. Whereas ceramide and sphingosine are associated with cellular growth arrest and apoptosis, S1P is associated with cellular survival and suppression of apoptosis [11]. Ceramide is broken down by ceramidases to sphingosine, which in turn is phosphorylated by one of two SphKs, SphK1 and SphK2, to generate S1P [12]. S1P can then either be dephosphorylated by two S1P-specific phosphatases (SPP1 and SPP2) or irreversibly degraded by S1P lyase (SPL) to phosphoethanolamine and hexadecenal [6, 12]. SphK1 is located close to the cell membrane, where it can be activated by numerous stimuli, including proinflammatory cytokines, to generate S1P [6]. Ceramide is also phosphorylated in the Golgi apparatus by ceramide kinase to produce ceramide-1-phosphate (C1P). These sphingolipid metabolites, ceramide, C1P, and S1P, are bioactive molecules which are important in inflammation. S1P is particularly important in immune cell trafficking [13]. There has been extensive investigation into the extracellular signaling of S1P, particularly its role in innate and adaptive immunity. We have learned much less about the intracellular targets and signaling of S1P.

It has been proposed that S1P formed by SphK1 in response to tumor-necrosis factor (TNF) binds to the TNF receptor-associated factor 2 (TRAF2) and enhances its E3 ligase activity. This leads to lysine-63-linked polyubiquitination of receptor interacting protein 1 (RIP1) and eventually NF-*κ*B activation [14]. TRAF-interacting protein (TRIP) suppresses the TRAF2 ubiquitin-dependent pathway by modulating the TRAF2-S1P interaction [15]. Within sites of sterile inflammation, S1P formed by SphK1 binds to the cellular inhibitor of apoptosis 2 (cIAP2) in response to interleukin-1 (IL-1) and enhances its lysine-63-linked polyubiquitination activities [16]. In response to IL-1, SphK1 and cIAP2 form a complex with interferon-regulatory factor 1 (IRF1), leading to its polyubiquitylation and activation. Consequently, IRF1 enhances expression of the chemokines CXCL10 and CCL5, which recruit mononuclear cells into sites of sterile inflammation [16]. Despite these findings, SphKs are not indispensable for the inflammatory response by macrophages [17]. This suggests that the role of SphKs as mediators in inflammatory cytokine signaling may be system or disease specific and not an essential part of the inflammatory cascade.

In contrast to the prosurvival SphK1, SphK2 inhibits cell growth and enhances apoptosis [18]. Furthermore, S1P formed in the nucleus by SphK2, or by inhibition of SPL, binds and inhibits the histone deacetylases HDAC1 and HDAC2, linking sphingolipid metabolism to inflammatory and metabolic gene expression [19, 20]. Interestingly, S1P produced in the mitochondria by SphK2 binds with high affinity and specificity to prohibitin 2 (PHB2), a highly conserved protein that regulates mitochondrial assembly and function [21]. Conjugated bile acids also bind to S1PR2 in hepatocytes [22] and the SphK2 generated S1P regulates hepatic lipid metabolism via histone deacetylase inhibition in the nucleus. This provides evidence for the role of S1P in the development of nonalcoholic fatty liver disease [23]. On the other hand, SphK1 was also reported to possess potential anti-inflammatory function by activation of p38 MAPK that suppress chemokine levels, and, in this system, activation of NF-*κ*B is separated from SphK1 [24]. Further, the neuroinflammatory response was significantly upregulated in LPS-induced brain injury in SphK1^−/−^ mice [25]. The function of SphKs and S1PR signaling in inflammation is still unclear and may be more complex than the current dogma.

Following transport out of cells, S1P binds to its ligand, consisting of a family of five specific G protein-coupled receptors in a paracrine and/or autocrine manner, known as “inside-out signaling” [1, 2, 11, 14, 16]. The crystal structure of S1PR1 suggests that extracellular access to the binding pocket by S1P occurs by sliding in the plane of membrane [26]. S1P regulates lymphocyte trafficking in immunity and allergy by attracting the lymphocytes to migrate via various receptors [27]. S1PR1 induces chemotaxis and membrane ruffling in phosphoinositide (PI) 3-kinase- and Rac-dependent manners, which induces a biphasic increase in the amount of the GTP-bound Rac. This causes the formation of the stress fibers and cytoskeletal rearrangement that decreases vascular permeability. S1PR1 and S1PR3 induce a migratory response in various types of immune cells. S1PR2 has been thought to possess function opposite of S1PR1 and S1PR3. As a G protein-coupled receptor, S1PR2 couples to Gi/o, Gq, and G12, and G13, as opposed to S1PR1, which couples solely to Gi/o. Activation of G12 and G13 leads to activation of Rho. S1PR2 has been associated with abolishment of IGF 1-directed chemotaxis and membrane ruffling, thus increasing vascular permeability in a manner dependent on the concentration gradient of S1P [28].

Recently, bile acids were found to bind to S1PR2 and regulate lipid metabolism in hepatocytes [23]. S1PR3 signaling in endothelial cells contributes to vasorelaxation. On the other hand, S1PR3 signaling in vascular smooth muscle cells contributes to vasopressor effect. Through such mechanisms, S1P and its analogues can influence heart rate via S1PR3 [29]. S1PR4 and S1PR5 have limited, specialized function in inflammation. S1PR4 is related to the migration of neutrophils from blood to tissue [30]. S1PR5 is expressed predominantly by oligodendrocytes and/or fibrous astrocytes in the rat brain and couples with Gi/o*α* proteins for migration and survival of those cells [31–33]. Patrolling monocytes also express high levels of S1PR5 similar to Natural Killer (NK) cells; however, it is suggested that S1PR5 in monocytes regulate their trafficking via a mechanism independent of S1P gradients [34]. S1P transport and extracellular signaling are an area of active research as they have implications for the tumor microenvironment in cancer and immune cell trafficking [2].

## 3. Role of S1P and S1PRs in the Regulation of Immune Cell Trafficking

S1P signaling via S1PRs is involved in various aspects of inflammatory cell function. T and B lymphocytes, as well as endothelial cells, express distinctive profiles of S1PRs. These S1PR profiles are major regulators of development, recirculation, tissue homing patterns, and chemotactic responses to chemokines of B and T cells [35]. S1PR signaling is also involved in modulation of circulating monocytes similar to lymphocytes and affects monocyte activation through CD40 expression and TNF-*α* production [36]. Notably, S1P regulates migration and endocytosis of mature dendritic cells via S1PR3, but not S1PR1 [37]. S1P increases macrophage homing, lymphocyte contact, and endothelial junctional complex formation in lymph nodes (LN) [38]. S1P mediates chemotaxis of macrophages* in vitro* and* in vivo* via S1PR3 and causes atherosclerosis by promoting inflammatory macrophage recruitment and altering smooth muscle cell behavior [10]. S1P is also involved in mast cell and eosinophil and dendritic cell recruitment in asthma [39].

Both the S1P gradient between the bone marrow and blood and the expression of S1PR1 are essential for optimal hematopoietic stem cell mobilization and trafficking during steady-state hematopoiesis [40]. During the inflammatory process, both S1PR expression on lymphocytes and endothelial cells and S1P levels in various immune compartments are modified. This results in transient arrest of lymphocytes in secondary lymphoid tissues, which is crucial for the generation of adaptive immunity and subsequent promotion of lymphocyte recruitment to sites of inflammation [29].

### 3.1. S1P-S1PR1 Axis in Lymphocyte Trafficking and Retention in Inflamed Tissue

Separate sources provide S1P to blood and lymphatic fluid [41]. Circulating blood S1P is believed to be mainly hematopoietic in origin, with erythrocytes as a major contributor, whereas lymphatic fluid S1P is from lymphatic endothelial cells. Recent studies clarified that hepatic apolipoprotein M (ApoM) produced by the liver increases S1P biosynthesis in hepatocytes and also influences plasma S1P levels [42, 43]. The majority of plasma S1P binds to ApoM in high-density lipoprotein (HDL). In spite of the fact that ApoM-S1P is not essential for lymphocyte trafficking, it inhibits lymphopoiesis through S1PR1 signaling in bone marrow lymphocyte progenitors [44].

**Figure 1:**
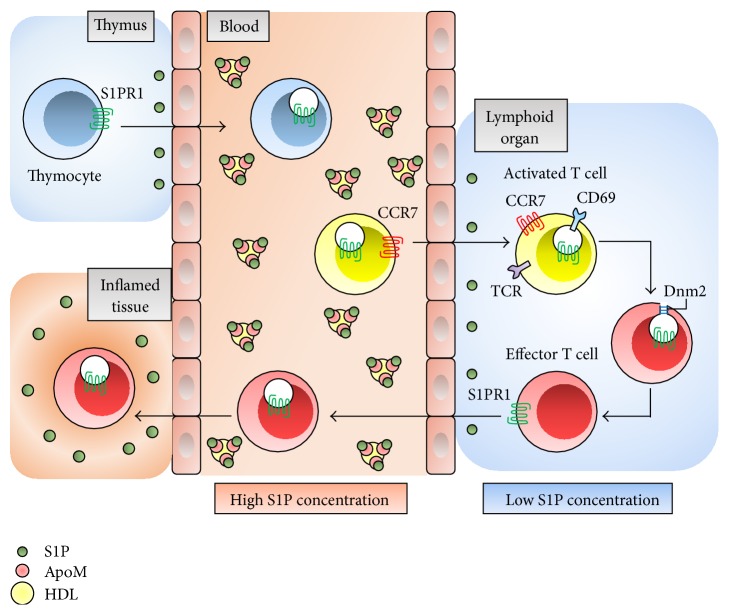
The role of S1P-S1PR1 axis in T cell trafficking. S1P is maintained at low concentration in the thymus and lymphoid organs and is in high concentration in blood, binding to ApoM in HDL particles. S1PR1 expression in T cells is downregulated in blood, and T cells are shifted into lymphoid organs with CCR7 signaling. CD69 forms a complex with S1PR1 and downregulates S1PR1 to promote retention in activated T cells. Because Dnm2 enables continuous S1PR1 signaling in lymphocytes, effector T cells can sense low S1P condition and egress from lymphoid organs. ApoM: apolipoprotein M; HDL: high-density lipoprotein; CCR7: CC-chemokine receptor 7; TCR: T cell receptor; Dnm2: dynamin 2.

The differential S1P concentration gradient facilitates egress of lymphocytes from lymphoid organs into blood and lymphatic fluid [13, 45]. In addition to the S1P gradient, S1PR1 is also essential for lymphocyte egress from the thymus and secondary lymphoid organs [46]. The positive gradient of S1P concentration between secondary lymphoid organs and lymphatic fluid presumably promotes S1PR1-dependent movement of T cells from secondary lymphoid organs back into the lymphatic circulation and then into blood [47]. Dynamin 2 is essential for S1PR1 internalization in low S1P concentrations and enables uninterrupted S1PR1 signaling and promotes S1P egress from both the thymus and LN. This function may be involved in the mechanism by which T cells sense low S1P concentrations and egress into circulatory fluids [48] (Figure 1).

Multiple S1PRs have been shown to be associated with lymphocyte biology, recirculation, and determination of T cell phenotypes. The expression of S1PR1 on T cells regulates their egress from the thymus and entry into the blood [49]. Lymphocyte S1PR1 expression is downregulated in the blood, upregulated in lymphoid organs, and downregulated again in the lymphatic fluid. This ligand-induced modulation of S1PR1 in circulating lymphocytes contributes to establishing their lymphoid organ transit time [50].

T cell activation and proliferation are mediated by the T cell antigen receptor (TCR), which translocates plasma membrane S1PR1 to the nuclear envelope membranes to facilitate association with Gi/o, Erk1/2, and other proteins [51]. T cells switch to a state favoring egress over retention by simultaneously upregulating S1PR1 and downregulating CCR7. LN retention of naïve lymphocytes depends on fibroblastic reticular cells (FRCs) of LN, while activated T cells remain in LN because of downregulated S1PR1 and are independent in FRCs [52]. CD69 can additionally form a complex with S1PR1 and downregulate S1PR1 through downstream IFN-*α*/IFN-*β*, and possibly other activating stimuli, to promote lymphocyte retention in lymphoid organs [53]. On the other hand, the S1P/S1PR2 axis inhibits early airway T cell recruitment in mast cell-dependent acute allergic responses in mice [54].

**Figure 2:**
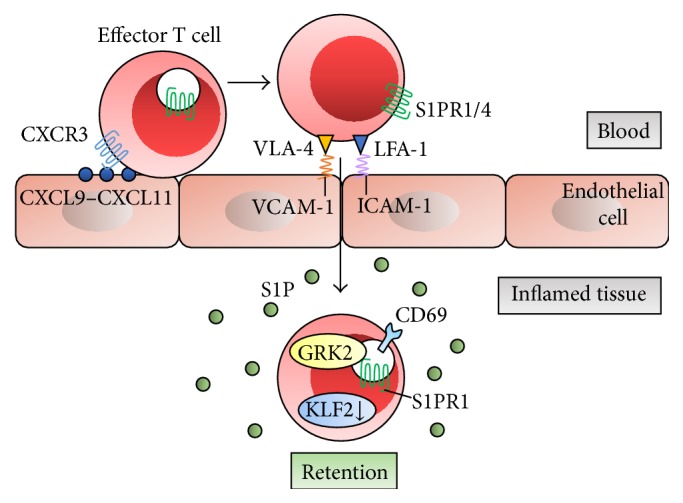
S1P-S1PR1 axis and lymphocyte retention in inflamed tissue. Surface expression of S1PR1 and S1PR4 on T cells is induced by chemokines CXCL9−CXCL11 presented on the endothelial surface and results in T cell migration into inflamed tissue. This is mediated at least partially by interactions of adhesion molecules LFA-1/ICAM-1 and VLA-4/VCAM-1. GRK2 downregulates S1PR1, allowing them to be retained in inflamed tissues. Downregulation of transcription factor KLF2 and S1PR1 transcription provides T cell retention in tissue, which is associated by early CD69 expression. CXCL: C-X-C chemokine ligand; CXCR3; C-X-C chemokine receptor 3; LFA-1: lymphocyte function-associated antigen-1; ICAM-1: intercellular adhesion molecule-1; VLA-4: very late antigen-4; VCAM-1: vascular cell adhesion molecule-1; GRK2: guanine nucleotide-binding protein-coupled receptor kinase-2; KLF2: Krüppel-like factor 2.

The increased S1P present in inflamed peripheral tissues may induce T cell retention. T cell migration from blood into tissue is induced by chemokines CXCL9–CXCL11 presented on the endothelial surface, which activates b1- and b2-integrin adhesion molecules and surface expression of S1PR1 and S1PR4 on T cells [55]. S1PR1 agonism inhibits migration of tissue T cells into afferent lymphatics in homeostatic and inflammatory conditions and causes the arrest of egress into inflamed tissues from the blood. This is mediated at least partially by interactions of the integrin LFA-1 with its ligand ICAM-1, and the integrin VLA-4 with its ligand VCAM-1 at the basal surface of lymphatic endothelium [56]. Heterotrimeric guanine nucleotide-binding protein-coupled receptor kinase-2 (GRK2) has been shown to function in downregulating S1PR1 on blood-exposed lymphocytes, allowing them to be retained in inflamed tissues [57]. According to the latest findings, regulation of KLF2 and S1PR1 transcription is associated with early CD69 expression and dictates whether CD8^+^ T cell recirculates or resides in the tissue [58] (Figure 2). CD69 interferes with S1PR function and regulates T cell retention and local memory formation [59]. On endothelial cells, B cell-derived peptide (PEPITEM) binds cadherin-15, promoting synthesis and release of S1P, thereby regulating T cell trafficking during inflammation and in response to adiponectin [55].

Activity of SPL, which metabolizes S1P, has been demonstrated to partially regulate S1P gradient-mediated lymphocyte trafficking [60, 61]. CD68^+^ cells on the parenchymal side of marginal reticular cells express SPL in human LN [62]. Inhibition of SPL by caramel food colorant, 2-acetyl-4-tetrahydroxybutylimidazole (THI), also prevents T cell egress from the thymus and secondary lymphoid organs under conditions of vitamin B6 deficiency [63].

B lymphocyte egress from secondary lymphoid organs also requires S1P and S1PR1. S1PR1 provides necessary signals for the transfer of newly generated immature B cells from the bone marrow to the blood [64, 65]. Marginal zone B cell localization to the marginal zone is regulated by response to the blood S1P, with S1PR1 signaling overcoming the recruiting activity of CXCL13 [66]. Marginal zone B cells migrate continually between the marginal zone and follicles, establishing the marginal zone as a site of S1PR1-dependent B cell egress from the follicles [67]. On the other hand, S1PR1 antagonism blocks passage through the cortical lymphatic endothelium and argues against a functional role for S1P gradient chemotaxis in B lymphocyte egress [68]. Overexpression of S1PR2 promotes the centering of activated B cells in the follicle and inhibits germinal center B cell responses to follicular chemoattractants and helps confine it to the germinal center [69]. S1PR2 suppresses growth and promotes local confinement of germinal center B cells through the G*α*13-dependent pathway [70]. Combinations of S1P receptors are different in various B cell populations and regulate the circulation of human B cell subsets. In human B cells, S1PR1-induced signaling istransmitted through *β*-arrestin 2, LPS-responsive beige-like anchor protein, dedicator of cytokinesis 8, and Wiskott-Aldrich syndrome protein [71].

### 3.2. S1P-S1PR5 Axis and Recruitment of NK Cells

Messenger RNA for S1PR1, S1PR4, and S1PR5, but not S1PR3, are expressed in NK cells [72]. S1P-deficient mice exhibit increased NK cell retention with inhibition of egress, indicating that while NK cells can develop within the thymus without S1PR1 expression, they are not retained in the peripheral tissue [73]. S1PR5 has also been shown to be required for NK cell egress from LN and bone marrow [74], and S1PR5-deficient mice have been reported to have aberrant NK cell homing during steady-state conditions. S1PR5 is also required for the mobilization of NK cells to inflamed tissues [75]. CD56^bright^ NK cells, a minority population of NK cells, express CCR7, and S1P influences the population, phenotype, and function of NK cells in peripheral circulation [76].

### 3.3. Contribution of Spns2 to Lymphocyte Trafficking

Spns2, which is a member of the major facilitator superfamily of non-ATP-dependent transporters, has been identified as a transporter of S1P in some cell types [77, 78]. S1P cannot spontaneously traverse the cell membrane lipid bilayer due to its polar head group and is secreted by either Spns2 or promiscuous ABC transporters [2, 79]. In breast cancer, multidrug resistant proteins ATP-binding cassette transporters, ABCC1 and ABCG2, export S1P after estrogen stimulation of breast cancer cells [79]. Spns2 is involved in angiogenesis, lymphangiogenesis, and the generation of the lymphatic network in LN during development [80]. Although it was initially assumed that the S1P gradient between the thymus and blood is the primary determinant of egress of mature T cells from the thymus, blood S1P level alone is insufficient to promote the egress [41, 80–82]. Spns2 plays a role in the regulation of S1P levels not only in the blood, but also in LN and lymphatic fluid, thus influencing lymphocyte trafficking and development of the lymphatic vessel network [80]. The immunological phenotype of Spns2 knockout mice closely mimics the phenotype of partial S1P deficiency, including impaired S1P-dependent lymphocyte trafficking, depletion of lymphocytes in the circulation, an increase in mature single-positive T cells in the thymus, and a selective reduction in mature B cells in the spleen and bone marrow, resulting in redistribution of lymphocytes from the spleen to LN [83]. This is consistent with the notion that normal egress from the spleen is due to blood S1P gradient, and blocked egress from LN is due to lymphatic fluid S1P gradient. Spns2 is needed in endothelial cells to supply lymphatic fluid S1P and support lymphocyte circulation [84]. Spns2 is currently believed to contribute to the S1P gradient required for T and B cells to egress from their respective primary lymphoid organs into lymphatic endothelial cells [85] (Figure 2). In agreement with this notion, we have recently found that Spns2-mediated S1P transport plays a significant role in the initiation and development of adaptive immune-related disorders and autoimmune diseases, such as asthma, colitis, multiple sclerosis, and arthritis in animal models [86].

### 3.4. FTY720 and Lymphopenia

FTY720 is a prodrug that acts as an immunomodulator after activation [4]. FTY720 was discovered by the chemical modification of the natural product, myriocin (ISP-1), which is a metabolite of the fungus* Isaria sinclairii*. Later, FTY720 was found to be a structural analogue of sphingosine and a functional antagonist of S1PRs [87]. Use of FTY720 has revealed that S1P is involved in lymphocyte egress from the thymus and secondary lymph organs into the circulation [88]. FTY720 can be administered orally and is approved by the United States Food and Drug Administration as a new treatment for multiple sclerosis, the most common inflammatory disorder of the central nervous system [89].

FTY720 is phosphorylated* in vivo* by SphKs to generate phosphorylated-FTY720 (p-FTY720), S1P mimetic which acts as a ligand for all of the S1PRs except S1PR2. p-FTY720 modulates chemotactic responses and lymphocyte trafficking by internalization of the S1PRs [6], thus strongly suppressing lymphocyte egress from the thymus and secondary lymphoid organs [90]. As S1P mimetic, p-FTY720 is also transported by Spns2 through the same pathway as S1P [91]. S1PR1 activated by p-FTY720 maintains signaling activity for several hours despite quantitative internalization. This sustained intracellular agonism may be an important mechanism that distinguishes FTY720 from other S1PR antagonists and contributes to the therapeutic potential of FTY720 [92]. p-FTY720 causes continued cAMP signaling that is not dependent on S1P1R redistribution and induces functional antagonism of Ca^2+^ signaling after transient stimulation [93].

After binding to S1PR1 and internalization into cells, S1P returns to the plasma membrane and is recycled within several hours. However, S1PR1 internalized by p-FTY720 does not lead to receptor recycling, and p-FTY720 strongly induces subsequent polyubiquitination and proteasomal degradation of the S1PR1 [94, 95]. The mechanism of S1PR1 internalization and modulation of autoimmune inflammation remains unclear. It was recently reported that incomplete S1PR1 phosphorylation worsens Th17-mediated autoimmune neuroinflammation, and this mechanism may be related to the pathogenesis of multiple sclerosis [96]. FTY720-induced S1PR1 internalization in T cells is caused by clathrin-mediated endocytosis and is regulated by moesin, an ezrin-radixin-moesin (ERM) family member [97].

S1PR1 suppression by FTY720 correlates with reduced numbers of lymphocytes and monocytes in experimental autoimmune encephalomyelitis in mice and rats independent of S1PR3 [36]. The percentages of central memory T cells (T_CM_) and naïve T cells decrease, while those of effector memory T cells (T_EM_) and suppressor precursor T cells (T_SP_) increase in both CD4^+^ T and CD8^+^ T cells with FTY720 therapy. The percentages of regulatory T cells (T_reg_) in CD4^+^ T cells and T_EM_ in CD8^+^ T cells also increase [98]. FTY720 can impair CD8^+^ T cell function independently of S1P pathway [99]. On the other hand, absolute numbers of NK cells are unchanged in FTY720-treated multiple sclerosis patients. However, relative proportions of NK cells within the whole circulating lymphoid population are increased. FTY720 causes a relative decrease in CD56^bright^ NK cells expressing CCR7, increased sensitivity to chemokine ligand, and promotes movement into LN [76]. In addition, FTY720 has nonimmunological mechanisms in astrocytes, which present S1P signaling pathways within the central nervous system as targets for multiple sclerosis therapies [100]. Finally, we have recently reported that p-FTY720 is a histone deacetylase inhibitor that reactivates estrogen receptor expression in breast cancer both* in vitro* and* in vivo*, suggesting that FTY720 may possess functions more than those that have previously been published [101]. More elucidation of the differences in functional mechanism between FTY720 and other S1P/S1PR modifying compounds will contribute to the investigation of S1P and the therapeutic potential of such compounds.

## 4. Role of S1P and S1PR1 in Lymphocyte Differentiation

In addition to trafficking, S1PR1 is also involved in lymphocyte differentiation. S1PR1 delivers intrinsic negative feedback to decrease thymic production and suppress activity of CD4^+^CD25^+^  T_reg_. S1PR1 blocks the differentiation of thymic T_reg_ precursors and inhibits the function of mature T_reg_ cells, thereby regulating T_reg_ cell-mediated immune tolerance [102]. S1PR1 signaling in T cells promotes tumor growth by inducing T_reg_ accumulation in tumors via STAT3 and inhibiting CD8^+^ T cell recruitment and activation [103]. FTY720 induces a decrease in circulating CD4^+^ T cells and CD19^+^ B cells while CD39^+^  T_reg_ cells increase in multiple sclerosis patients [104]. FTY720 directly potentiates recruitment and function of myeloid-derived suppresser cells (MDSCs) and controls the differentiation of Th1 cells to T_reg_ by targeting S1PR1 [105]. The effect of S1P in lymphocyte differentiation is related to the immune response against cancer and pathogenesis of autoimmune diseases. Further investigation and therapeutic application are expected in the near future.

## 5. Therapeutic Potential through Targeting Local S1P/S1PR Function in Inflamed Tissues

### 5.1. Asthma

ORM- (yeast-) like protein isoform 3 (ORMDL3), which is identified as a gene associated with susceptibility to asthma, promotes eosinophil trafficking, recruitment, and activation [106] and regulates sphingolipid and ceramide homeostasis [107]. Intranasal application of FTY720 was shown to decrease ORMDL3 expression and is effective for reducing airway inflammation and hyperreactivity and mucus hypersecretion in house dust mite-challenged mice [108]. On the other hand, it has been reported that prolonged FTY720 treatment induces life-threatening asthma attacks and deterioration [109]. Further investigations of therapeutic effects of FTY720 or other S1P/S1PR related-compounds for asthma diseases are expected.

### 5.2. Allergic Rhinitis

Allergic rhinitis and asthma are the two most common allergic diseases [110]. Intranasal FTY720 treatment significantly decreases eosinophils, mast cells, and dendritic cells in the nasal mucosa of animal allergic rhinitis models with decreased levels of IL-4, IL-5, IL-10, and IL-13 in LN of FTY720-treated animals. The mechanism includes impairment of Th2 differentiation and proliferation, inhibition of eosinophilia, and induction of apoptosis in mast cells [111].

### 5.3. Allergic Skin Diseases and Psoriasis

S1P controls several fundamental functions of keratinocytes and skin dendritic cells. S1P suppresses proliferation and promotes differentiation of keratinocytes. Antigen uptake, migration, and cytokine production in dendritic cells are regulated by sphingolipids. Dysregulation of sphingolipid metabolism is involved in inflammatory skin diseases such as atopic dermatitis [112]. Topical administration of S1P or FTY720 inhibits dendritic cell migration and regulates Langerhans cell migration from skin to LN and is an effective treatment for allergic skin diseases such as contact hypersensitivity and atopic dermatitis [113]. Although genetic factors, epithelial disorders, and environmental factors are involved in the pathogenesis of psoriasis, inflammation is also implicated in the progression of psoriasis. Topical administration of S1P and FTY720 has been reported to be effective for psoriasis [114]. Ponesimod, a selective S1PR1 modulator, is a functional antagonist of S1PR1, and its oral administration is undergoing clinical trial for psoriasis [115]. Considering that there are various clinical phenotypes of psoriasis, topical therapies targeting S1P/S1PR function might be a new option for the control of mild-to-moderate psoriasis lesions.

### 5.4. Corneal Allograft

Corneal transplantation is the most common and successful solid organ transplantation. Despite the fact that HLA matching and systemic immunosuppression are not regularly utilized, 90% of first-time corneal allografts succeed [116]. However, in order to achieve even better outcomes, there remains the option of topical administration of immunosuppressive medication. Treatment with FTY720 eye-drops can effectively prolong allogeneic corneal graft survival in mice. Topical application of FTY720 increases the percentage of CD4^+^ T cells and T_reg_ in cervical LN, increases TGF-*β*1 mRNA expression, and decreases infiltration of CD4^+^ T cells in corneal allografts [117]. Corneal graft survival is prolonged by topical application of S1PR1, and S1PR1 selective agonist may be effective in the inhibition of corneal allograft rejection [118, 119].

### 5.5. Wound Healing

Wound healing is one of the most fundamental research topics in surgery, since every surgical intervention creates wounds. The stages of wound healing are classified into three phases: inflammatory, proliferative, and remodeling phases [120]. The inflammatory phase is the first process of wound healing during which purification of the wound and production of cytokines and chemokines by inflammatory cells occur. The inflammatory phase strongly influences the following phases, as discovered through complications such as intractable wounds and abnormal scars, termed hypertrophic scars and keloid formation. Thus, strengthening of the inflammatory reaction by activation of S1P signaling is expected to promote wound healing. In addition, S1P promotes formation of fibronectin matrix at the dermal-epidermal junction, and keratinocyte migration, which is expected to promote wound healing. Further, in response to injury, thrombin promotes the activation of S1P, which promotes angiogenesis for wound healing [121]. Direct SphK1 plasmid application to wounds was shown to accelerate wound closure in diabetic rats [122]. This warrants further detailed analysis and investigation as human wounds heal differently from other mammals. For instance, wound healing takes longer in humans and often results in hypertrophic scar or keloid, which is rarely observed in other mammals. Treatments to promote wound healing, which are currently limited to modifying nutrition and circulation, are expected to have a large potential impact on all phases of health recovery. S1P may be an ideal target molecule to promote wound healing.

## 6. Conclusion

S1P is a bioactive lipid mediator that is increasingly recognized as an important regulator of immune function. S1PR expression and S1P concentration gradient have been implicated in immune cell development, differentiation, and recruitment during both acute and chronic inflammation. Currently, numerous studies are in progress to investigate the possibility of new therapies targeting S1P signaling, including FTY720, which may have great potential as a therapeutic target for many types of diseases such as autoimmune diseases, allergy, infection, and chronic inflammation. A large number of positive results thus far support the development of S1P signaling targeted therapies to treat such conditions.

## References

[1] Takabe K., Paugh S. W., Milstien S., Spiegel S. (2008). ‘Inside-out’ signaling of sphingosine-1-phosphate: therapeutic targets. *Pharmacological Reviews*.

[2] Takabe K., Spiegel S. (2014). Export of sphingosine-1-phosphate and cancer progression. *Journal of Lipid Research*.

[3] Nagahashi M., Ramachandran S., Kim E. Y. (2012). Sphingosine-1-phosphate produced by sphingosine kinase 1 promotes breast cancer progression by stimulating angiogenesis and lymphangiogenesis. *Cancer Research*.

[4] Proia R. L., Hla T. (2015). Emerging biology of sphingosine-1-phosphate: its role in pathogenesis and therapy. *The Journal of Clinical Investigation*.

[5] Takabe K., Yamada A., Rashid O. M. (2012). Twofer anti-vascular therapy targeting sphingosine-1-phosphate for breast cancer. *Gland Surgery*.

[6] Spiegel S., Milstien S. (2011). The outs and the ins of sphingosine-1-phosphate in immunity. *Nature Reviews Immunology*.

[7] Huang W., Nagahashi M., Terracina K., Takabe K. (2013). Emerging role of sphingosine-1-phosphate in inflammation, cancer, and lymphangiogenesis. *Biomolecules*.

[8] Bolli M. H., Abele S., Binkert C. (2010). 2-Imino-thiazolidin-4-one derivatives as potent, orally active S1P_1_ receptor agonists. *Journal of Medicinal Chemistry*.

[9] Maeda Y., Seki N., Kataoka H. (2015). IL-17-producing V*γ*4+ *γδ* T cells require sphingosine 1-phosphate receptor 1 for their egress from the lymph nodes under homeostatic and inflammatory conditions. *The Journal of Immunology*.

[10] Keul P., Lucke S., Von Wnuck Lipinski K. (2011). Sphingosine-1-phosphate receptor 3 promotes recruitment of monocyte/macrophages in inflammation and atherosclerosis. *Circulation Research*.

[11] Hait N. C., Oskeritzian C. A., Paugh S. W., Milstien S., Spiegel S. (2006). Sphingosine kinases, sphingosine 1-phosphate, apoptosis and diseases. *Biochimica et Biophysica Acta—Biomembranes*.

[12] Chalfant C. E., Spiegel S. (2005). Sphingosine 1-phosphate and ceramide 1-phosphate: expanding roles in cell signaling. *Journal of Cell Science*.

[13] Maceyka M., Spiegel S. (2014). Sphingolipid metabolites in inflammatory disease. *Nature*.

[14] Alvarez S. E., Harikumar K. B., Hait N. C. (2010). Sphingosine-1-phosphate is a missing cofactor for the E3 ubiquitin ligase TRAF2. *Nature*.

[15] Park E.-S., Choi S., Shin B. (2015). Tumor necrosis factor (TNF) receptor-associated factor (TRAF)-interacting protein (TRIP) negatively regulates the TRAF2 ubiquitin-dependent pathway by suppressing the TRAF2-sphingosine 1-phosphate (S1P) interaction. *The Journal of Biological Chemistry*.

[16] Harikumar K. B., Yester J. W., Surace M. J. (2014). K63-linked polyubiquitination of transcription factor IRF1 is essential for IL-1-induced production of chemokines CXCL10 and CCL5. *Nature Immunology*.

[17] Xiong Y., Lee H. J., Mariko B. (2013). Sphingosine kinases are not required for inflammatory responses in macrophages. *The Journal of Biological Chemistry*.

[18] Maceyka M., Sankala H., Hait N. C. (2005). SphK1 and SphK2, sphingosine kinase isoenzymes with opposing functions in sphingolipid metabolism. *The Journal of Biological Chemistry*.

[19] Hait N. C., Allegood J., Maceyka M. (2009). Regulation of histone acetylation in the nucleus by sphingosine-1-phosphate. *Science*.

[20] Nguyen-Tran D.-H., Hait N. C., Sperber H. (2014). Molecular mechanism of sphingosine-1-phosphate action in Duchenne muscular dystrophy. *DMM Disease Models and Mechanisms*.

[21] Strub G. M., Paillard M., Liang J. (2011). Sphingosine-1-phosphate produced by sphingosine kinase 2 in mitochondria interacts with prohibitin 2 to regulate complex IV assembly and respiration. *The FASEB Journal*.

[22] Studer E., Zhou X., Zhao R. (2012). Conjugated bile acids activate the sphingosine-1-phosphate receptor 2 in primary rodent hepatocytes. *Hepatology*.

[23] Nagahashi M., Takabe K., Liu R. (2015). Conjugated bile acid-activated S1P receptor 2 is a key regulator of sphingosine kinase 2 and hepatic gene expression. *Hepatology*.

[24] Adada M. M., Alexa Orr-Gandy K., Snider A. J. (2013). Sphingosine kinase 1 regulates tumor necrosis factor-mediated RANTES induction through p38 mitogen-activated protein kinase but independently of nuclear factor *κ*B activation. *The Journal of Biological Chemistry*.

[25] Grin'kina N. M., Karnabi E. E., Damania D., Wadgaonkar S., Muslimov I. A., Wadgaonkar R. (2012). Sphingosine kinase 1 deficiency exacerbates LPS-induced neuroinflammation. *PloS ONE*.

[26] Hanson M. A., Roth C. B., Jo E. (2012). Crystal structure of a lipid G protein-coupled receptor. *Science*.

[27] Spiegel S., Milstien S. (2007). Functions of the multifaceted family of sphingosine kinases and some close relatives. *The Journal of Biological Chemistry*.

[28] Okamoto H., Takuwa N., Yokomizo T. (2000). Inhibitory regulation of Rac activation, membrane ruffling, and cell migration by the G protein-coupled sphingosine-1-phosphate receptor EDG5 but not EDG1 or EDG3. *Molecular and Cellular Biology*.

[29] Swan D. J., Kirby J. A., Ali S. (2010). Vascular biology: the role of sphingosine 1-phosphate in both the resting state and inflammation. *Journal of Cellular and Molecular Medicine*.

[30] Allende M. L., Bektas M., Lee B. G. (2011). Sphingosine-1-phosphate lyase deficiency produces a pro-inflammatory response while impairing neutrophil trafficking. *The Journal of Biological Chemistry*.

[31] Im D.-S., Heise C. E., Ancellin N. (2000). Characterization of a novel sphingosine 1-phosphate receptor, Edg-8. *The Journal of Biological Chemistry*.

[32] Jaillard C., Harrison S., Stankoff B. (2005). Edg8/S1P5: an oligodendroglial receptor with dual function on process retraction and cell survival. *The Journal of Neuroscience*.

[33] Novgorodov A. S., El-Alwani M., Bielawski J., Obeid L. M., Gudz T. I. (2007). Activation of sphingosine-1-phosphate receptor S1P5 inhibits oligodendrocyte progenitor migration. *The FASEB Journal*.

[34] Debien E., Mayol K., Biajoux V. (2013). S1PR5 is pivotal for the homeostasis of patrolling monocytes. *European Journal of Immunology*.

[35] Goetzl E. J., Rosen H. (2004). Regulation of immunity by lysosphingolipids and their G protein-coupled receptors. *The Journal of Clinical Investigation*.

[36] Lewis N. D., Haxhinasto S. A., Anderson S. M. (2013). Circulating monocytes are reduced by sphingosine-1-phosphate receptor modulators independently of S1P_3_. *The Journal of Immunology*.

[37] Maeda Y., Matsuyuki H., Shimano K., Kataoka H., Sugahara K., Chiba K. (2007). Migration of CD4 T cells and dendritic cells toward sphingosine 1-phosphate (S1P) is mediated by different receptor subtypes: S1P regulates the functions of murine mature dendritic cells via S1P receptor type 3. *The Journal of Immunology*.

[38] Singer I. I., Tian M., Wickham L. A. (2005). Sphingosine-1-phosphate agonists increase macrophage homing, lymphocyte contacts, and endothelial junctional complex formation in murine lymph nodes. *The Journal of Immunology*.

[39] Price M. M., Oskeritzian C. A., Falanga Y. T. (2013). A specific sphingosine kinase 1 inhibitor attenuates airway hyperresponsiveness and inflammation in a mast cell-dependent murine model of allergic asthma. *Journal of Allergy and Clinical Immunology*.

[40] Juarez J. G., Harun N., Thien M. (2012). Sphingosine-1-phosphate facilitates trafficking of hematopoietic stem cells and their mobilization by CXCR4 antagonists in mice. *Blood*.

[41] Pappu R., Schwab S. R., Cornelissen I. (2007). Promotion of lymphocyte egress into blood and lymph by distinct sources of sphingosine-1-phosphate. *Science*.

[42] Kurano M., Tsukamoto K., Ohkawa R. (2013). Liver involvement in sphingosine 1-phosphate dynamism revealed byadenoviral hepatic overexpression of apolipoprotein M. *Atherosclerosis*.

[43] Liu M., Seo J., Allegood J. (2014). Hepatic apolipoprotein M (ApoM) overexpression stimulates formation of larger ApoM/sphingosine 1-phosphate-enriched plasma high density lipoprotein. *The Journal of Biological Chemistry*.

[44] Blaho V. A., Galvani S., Engelbrecht E. (2015). HDL-bound sphingosine-1-phosphate restrains lymphopoiesis and neuroinflammation. *Nature*.

[45] Schwab S. R., Cyster J. G. (2007). Finding a way out: lymphocyte egress from lymphoid organs. *Nature Immunology*.

[46] Rivera J., Proia R. L., Olivera A. (2008). The alliance of sphingosine-1-phosphate and its receptors in immunity. *Nature Reviews Immunology*.

[47] Rosen H., Goetzl E. J. (2005). Sphingosine 1-phosphate and its receptors: an autocrine and paracrine network. *Nature Reviews Immunology*.

[48] Willinger T., Ferguson S. M., Pereira J. P., De Camilli P., Flavell R. A. (2014). Dynamin 2-dependent endocytosis is required for sustained S1PR1 signaling. *The Journal of Experimental Medicine*.

[49] Garris C. S., Blaho V. A., Hla T., Han M. H. (2014). Sphingosine-1-phosphate receptor 1 signalling in T cells: trafficking and beyond. *Immunology*.

[50] Lo C. G., Xu Y., Proia R. L., Cyster J. G. (2005). Cyclical modulation of sphingosine-1-phosphate receptor 1 surface expression during lymphocyte recirculation and relationship to lymphoid organ transit. *The Journal of Experimental Medicine*.

[51] Liao J.-J., Huang M.-C., Graler M., Huang Y., Qiu H., Goetzl E. J. (2007). Distinctive T cell-suppressive signals from nuclearized type 1 sphingosine 1-phosphate G protein-coupled receptors. *The Journal of Biological Chemistry*.

[52] Denton A. E., Roberts E. W., Linterman M. A., Fearon D. T. (2014). Fibroblastic reticular cells of the lymph node are required for retention of resting but not activated CD8^+^ T cells. *Proceedings of the National Academy of Sciences of the United States of America*.

[53] Shiow L. R., Rosen D. B., Brdičková N. (2006). CD69 acts downstream of interferon-*α*/*β* to inhibit S1P_1_ and lymphocyte egress from lymphoid organs. *Nature*.

[54] Oskeritzian C. A., Hait N. C., Wedman P. (2014). The sphingosine-1-phosphate/sphingosine-1-phosphate receptor 2 axis regulates early airway T-cell infiltration in murine mast cell-dependent acute allergic responses. *Journal of Allergy and Clinical Immunology*.

[55] Chimen M., McGettrick H. M., Apta B. (2015). Homeostatic regulation of T cell trafficking by a B cell-derived peptide is impaired in autoimmune and chronic inflammatory disease. *Nature Medicine*.

[56] Ledgerwood L. G., Lal G., Zhang N. (2008). The sphingosine 1-phosphate receptor 1 causes tissue retention by inhibiting the entry of peripheral tissue T lymphocytes into afferent lymphatics. *Nature Immunology*.

[57] Arnon T. I., Xu Y., Lo C. (2011). GRK2-dependent S1PR1 desensitization is required for lymphocytes to overcome their attraction to blood. *Science*.

[58] Skon C. N., Lee J.-Y., Anderson K. G., Masopust D., Hogquist K. A., Jameson S. C. (2013). Transcriptional downregulation of *S1pr1* is required for the establishment of resident memory CD8^+^ T cells. *Nature Immunology*.

[59] Mackay L. K., Braun A., Macleod B. L. (2015). Cutting edge: CD69 interference with sphingosine-1-phosphate receptor function regulates peripheral T cell retention. *The Journal of Immunology*.

[60] Bot M., Van Veldhoven P. P., de Jager S. C. A. (2013). Hematopoietic sphingosine 1-phosphate lyase deficiency decreases atherosclerotic lesion development in LDL-receptor deficient mice. *PLoS ONE*.

[61] Schwab S. R., Pereira J. P., Matloubian M., Xu Y., Huang Y., Cyster J. G. (2005). Lymphocyte sequestration through S1P lyase inhibition and disruption of S1P gradients. *Science*.

[62] Park S. M., Angel C. E., Mcintosh J. D. (2014). Sphingosine-1-phosphate lyase is expressed by CD68^+^ cells on the parenchymal side of marginal reticular cells in human lymph nodes. *European Journal of Immunology*.

[63] Ohtoyo M., Tamura M., Machinaga N., Muro F., Hashimoto R. (2014). Sphingosine 1-phosphate lyase inhibition by 2-acetyl-4-(tetrahydroxybutyl)imidazole (THI) under conditions of vitamin B6 deficiency. *Molecular and Cellular Biochemistry*.

[64] Pereira J. P., Cyster J. G., Xu Y. (2010). A role for S1P and S1P1 in immature-B cell egress from mouse bone marrow. *PLoS ONE*.

[65] Allende M. L., Tuymetova G., Lee B. G., Bonifacino E., Wu Y.-P., Proia R. L. (2010). S1P1 receptor directs the release of immature B cells from bone marrow into blood. *The Journal of Experimental Medicine*.

[66] Cinamon G., Matloubian M., Lesneski M. J. (2004). Sphingosine 1-phosphate receptor 1 promotes B cell localization in the splenic marginal zone. *Nature Immunology*.

[67] Arnon T. I., Horton R. M., Grigorova I. L., Cyster J. G. (2013). Visualization of splenic marginal zone B-cell shuttling and follicular B-cell egress. *Nature*.

[68] Sinha R. K., Park C., Hwang I.-Y., Davis M. D., Kehrl J. H. (2009). B lymphocytes exit lymph nodes through cortical lymphatic sinusoids by a mechanism independent of sphingosine-1-phosphate-mediated chemotaxis. *Immunity*.

[69] Green J. A., Suzuki K., Cho B. (2011). The sphingosine 1-phosphate receptor S1P_2_ maintains the homeostasis of germinal center B cells and promotes niche confinement. *Nature Immunology*.

[70] Muppidi J. R., Schmitz R., Green J. A. (2014). Loss of signalling via G*α*13 in germinal centre B-cell-derived lymphoma. *Nature*.

[71] Sic H., Kraus H., Madl J. (2014). Sphingosine-1-phosphate receptors control B-cell migration through signaling components associated with primary immunodeficiencies, chronic lymphocytic leukemia, and multiple sclerosis. *Journal of Allergy and Clinical Immunology*.

[72] Kveberg L., Bryceson Y., Inngjerdingen M., Rolstad B., Maghazachi A. A. (2002). Sphingosine 1 phosphate induces the chemotaxis of human natural killer cells. Role for heterotrimeric G proteins and phosphoinositide 3 kinases. *European Journal of Immunology*.

[73] Allende M. L., Zhou D., Kalkofen D. N. (2008). S1P1 receptor expression regulates emergence of NKT cells in peripheral tissues. *The FASEB Journal*.

[74] Jenne C. N., Enders A., Rivera R. (2009). T-bet-dependent S1P_5_ expression in NK cells promotes egress from lymph nodes and bone marrow. *The Journal of Experimental Medicine*.

[75] Walzer T., Chiossone L., Chaix J. (2007). Natural killer cell trafficking in vivo requires a dedicated sphingosine 1-phosphate receptor. *Nature Immunology*.

[76] Johnson T. A., Evans B. L., Durafourt B. A. (2011). Reduction of the peripheral blood CD56^bright^ NK lymphocyte subset in FTY720-treated multiple sclerosis patients. *The Journal of Immunology*.

[77] Kawahara A., Nishi T., Hisano Y., Fukui H., Yamaguchi A., Mochizuki N. (2009). The sphingolipid transporter Spns2 functions in migration of zebrafish myocardial precursors. *Science*.

[78] Osborne N., Brand-Arzamendi K., Ober E. A. (2008). The spinster homolog, two of hearts, is required for sphingosine 1-phosphate signaling in zebrafish. *Current Biology*.

[79] Takabe K., Kim R. H., Allegood J. C. (2010). Estradiol induces export of sphingosine 1-phosphate from breast cancer cells via ABCC1 and ABCG2. *The Journal of Biological Chemistry*.

[80] Nagahashi M., Kim E. Y., Yamada A. (2013). Spns2, a transporter of phosphorylated sphingoid bases, regulates their blood and lymph levels, and the lymphatic network. *The FASEB Journal*.

[81] Zachariah M. A., Cyster J. G. (2010). Neural crest-derived pericytes promote egress of mature thymocytes at the corticomedullary junction. *Science*.

[82] Bréart B., Ramos-Perez W. D., Mendoza A. (2011). Lipid phosphate phosphatase 3 enables efficient thymic egress. *The Journal of Experimental Medicine*.

[83] Nijnik A., Clare S., Hale C. (2012). The role of sphingosine-1-phosphate transporter Spns2 in immune system function. *The Journal of Immunology*.

[84] Mendoza A., Bréart B., Ramos-Perez W. D. (2012). The transporter Spns2 is required for secretion of lymph but not plasma sphingosine-1-phosphate. *Cell Reports*.

[85] Fukuhara S., Simmons S., Kawamura S. (2012). The sphingosine-1-phosphate transporter Spns2 expressed on endothelial cells regulates lymphocyte trafficking in mice. *The Journal of Clinical Investigation*.

[86] Donoviel M. S., Hait N. C., Ramachandran S. (2015). Spinster 2, a sphingosine-1-phosphate transporter, plays a critical role in inflammatory and autoimmune diseases. *The FASEB Journal*.

[87] Brinkmann V., Billich A., Baumruker T. (2010). Fingolimod (FTY720): discovery and development of an oral drug to treat multiple sclerosis. *Nature Reviews Drug Discovery*.

[88] Mandala S., Hajdu R., Bergstrom J. (2002). Alteration of lymphocyte trafficking by sphingosine-1-phosphate receptor agonists. *Science*.

[89] Prager B., Spampinato S. F., Ransohoff R. M. (2015). Sphingosine 1-phosphate signaling at the blood–brain barrier. *Trends in Molecular Medicine*.

[90] Cyster J. G. (2005). Chemokines, sphingosine-1-phosphate, and cell migration in secondary lymphoid organs. *Annual Review of Immunology*.

[91] Hisano Y., Kobayashi N., Kawahara A., Yamaguchi A., Nishi T. (2011). The sphingosine 1-phosphate transporter, SPNS2, functions as a transporter of the phosphorylated form of the immunomodulating agent FTY720. *The Journal of Biological Chemistry*.

[92] Mullershausen F., Zecri F., Cetin C., Billich A., Guerini D., Seuwen K. (2009). Persistent signaling induced by FTY720-phosphate is mediated by internalized S1P1 receptors. *Nature Chemical Biology*.

[93] Healy L. M., Sheridan G. K., Pritchard A. J., Rutkowska A., Mullershausen F., Dev K. K. (2013). Pathway specific modulation of S1P1 receptor signalling in rat and human astrocytes. *British Journal of Pharmacology*.

[94] Oo M. L., Thangada S., Wu M.-T. (2007). Immunosuppressive and anti-angiogenic sphingosine 1-phosphate receptor-1 agonists induce ubiquitinylation and proteasomal degradation of the receptor. *The Journal of Biological Chemistry*.

[95] Sykes D. A., Riddy D. M., Stamp C. (2014). Investigating the molecular mechanisms through which FTY720-P causes persistent S1P_1_ receptor internalization. *British Journal of Pharmacology*.

[96] Garris C. S., Wu L., Acharya S. (2013). Defective sphingosine 1-phosphate receptor 1 (S1P_1_) phosphorylation exacerbates T_H_17-mediated autoimmune neuroinflammation. *Nature Immunology*.

[97] Nomachi A., Yoshinaga M., Liu J. (2013). Moesin controls clathrin-mediated S1PR1 internalization in T cells. *PLoS ONE*.

[98] Song Z.-Y., Yamasaki R., Kawano Y. (2015). Peripheral blood T cell dynamics predict relapse in multiple sclerosis patients on fingolimod. *PLoS ONE*.

[99] Ntranos A., Hall O., Robinson D. P. (2014). FTY720 impairs CD8 T-cell function independently of the sphingosine-1-phosphate pathway. *Journal of Neuroimmunology*.

[100] Choi J. W., Gardell S. E., Herr D. R. (2011). FTY720 (fingolimod) efficacy in an animal model of multiple sclerosis requires astrocyte sphingosine 1-phosphate receptor 1 (S1P_1_) modulation. *Proceedings of the National Academy of Sciences of the United States of America*.

[101] Hait N. C., Avni D., Yamada A. (2015). The phosphorylated prodrug FTY720 is a histone deacetylase inhibitor that reactivates ER*α* expression and enhances hormonal therapy for breast cancer. *Oncogenesis*.

[102] Liu G., Burns S., Huang G. (2009). The receptor S1P_1_ overrides regulatory T cell-mediated immune suppression through Akt-mTOR. *Nature Immunology*.

[103] Priceman S. J., Shen S., Wang L. (2014). S1PR1 is crucial for accumulation of regulatory T cells in tumors via STAT3. *Cell Reports*.

[104] Muls N., Dang H. A., Sindic C. J. M., van Pesch V. (2014). Fingolimod increases CD39-expressing regulatory T cells in multiple sclerosis patients. *PLoS ONE*.

[105] Liu G., Bi Y., Wang R. (2014). Targeting S1P1 receptor protects against murine immunological hepatic injury through myeloid-derived suppressor cells. *The Journal of Immunology*.

[106] Ha S. G., Ge X. N., Bahaie N. S. (2013). ORMDL3 promotes eosinophil trafficking and activation via regulation of integrins and CD48. *Nature Communications*.

[107] Breslow D. K., Collins S. R., Bodenmiller B. (2010). Orm family proteins mediate sphingolipid homeostasis. *Nature*.

[108] Oyeniran C., Sturgill J. L., Hait N. C. (2015). Aberrant ORM (yeast)–like protein isoform 3 (ORMDL3) expression dysregulates ceramide homeostasis in cells and ceramide exacerbates allergic asthma in mice. *Journal of Allergy and Clinical Immunology*.

[109] Zecca C., Caporro M., Györik S., Gobbi C. (2014). Life-threatening asthma attack during prolonged fingolimod treatment: case report. *Patient Preference and Adherence*.

[110] Bender B. G. (2015). Motivating patient adherence to allergic rhinitis treatments. *Current Allergy and Asthma Reports*.

[111] Kleinjan A., van Nimwegen M., Leman K., Hoogsteden H. C., Lambrecht B. N. (2013). Topical treatment targeting sphingosine-1-phosphate and sphingosine lyase abrogates experimental allergic rhinitis in a murine model. *Allergy*.

[112] Japtok L., Bäumer W., Kleuser B. (2014). Sphingosine-1-phosphate as signaling molecule in the skin. *Allergo Journal International*.

[113] Reines I., Kietzmann M., Mischke R. (2009). Topical application of sphingosine-1-phosphate and FTY720 attenuate allergic contact dermatitis reaction through inhibition of dendritic cell migration. *The Journal of Investigative Dermatology*.

[114] Schaper K., Dickhaut J., Japtok L. (2013). Sphingosine-1-phosphate exhibits anti-proliferative and anti-inflammatory effects in mouse models of psoriasis. *Journal of Dermatological Science*.

[115] Vaclavkova A., Chimenti S., Arenberger P. (2014). Oral ponesimod in patients with chronic plaque psoriasis: a randomised, double-blind, placebo-controlled phase 2 trial. *The Lancet*.

[116] Niederkorn J. Y. (2015). Immunology of corneal allografts: insights from animal models. *Journal of Clinical and Experimental Ophthalmology*.

[117] Liu Y., Jiang J., Xiao H. (2012). Topical application of FTY720 and cyclosporin A prolong corneal graft survival in mice. *Molecular Vision*.

[118] Jia L., Liu Y., Wang L., Zhu J., Huang Y. (2014). Effects of topical sphingosine-1-phosphate 1 receptor agonist on corneal allograft in mice. *Cornea*.

[119] Zhu J., Liu Y., Huang Y. (2015). Topical application of sphingosine 1-phosphate receptor 1 prolongs corneal graft survival in mice. *Molecular Medicine Reports*.

[120] Stein C., Küchler S. (2013). Targeting inflammation and wound healing by opioids. *Trends in Pharmacological Sciences*.

[121] Watterson K. R., Lanning D. A., Diegelmann R. F., Spiegel S. (2007). Regulation of fibroblast functions by lysophospholipid mediators: potential roles in wound healing. *Wound Repair and Regeneration*.

[122] Yu H., Yuan L., Xu M., Zhang Z., Duan H. (2014). Sphingosine kinase 1 improves cutaneous wound healing in diabetic rats. *Injury*.

