# Corrigendum to “Sphingosine-1-Phosphate Signaling in Immune Cells and Inflammation: Roles and Therapeutic Potential”

**DOI:** 10.1155/2016/2856829

**Published:** 2016-10-24

**Authors:** Masayo Aoki, Hiroaki Aoki, Rajesh Ramanathan, Nitai C. Hait, Kazuaki Takabe

**Affiliations:** ^1^Division of Surgical Oncology, Department of Surgery, Virginia Commonwealth University School of Medicine and Massey Cancer Center, West Hospital 7-402, 1200 East Broad Street, P.O. Box 980011, Richmond, VA 23298-0011, USA; ^2^Department of Biochemistry & Molecular Biology, Virginia Commonwealth University School of Medicine and Massey Cancer Center, West Hospital 7-402, 1200 East Broad Street, P.O. Box 980011, Richmond, VA 23298-0011, USA; ^3^Breast Surgery, Roswell Park Cancer Institute, Elm & Carlton Streets, Buffalo, NY 14263, USA

In the article titled “Sphingosine-1-Phosphate Signaling in Immune Cells and Inflammation: Roles and Therapeutic Potential” [1], there was an error in the “FTY720 and Lymphopenia” section, where the sentence “p-FTY720 causes continued cAMP signaling that is not dependent on S1P1R redistribution and induces functional antagonism of Ca^2+^ signaling after transient stimulation” should be corrected to “p-FTY720 causes continued cAMP signaling that is not dependent on S1PR1 redistribution and induces functional antagonism of Ca^2+^ signaling after transient stimulation.”
